# Supplementary material for: Spatial and temporal invasion dynamics of the 2014–2017 Zika and chikungunya epidemics in Colombia
Source: PLoS Comput Biol. 2021 Jul 2;17(7):e1009174. doi: 10.1371/journal.pcbi.1009174 (PMC8291727; doi:10.1371/journal.pcbi.1009174)
Supplement: S1 Text — Fig A. Weekly reported cases of chikungunya fever (CF), dengue fever (DF), and Zika virus disease (ZVD) in Colombia, January 2010 –June 2017. Fig B. Epidemiological curves of chikungunya fever (CF) and Zika virus disease (ZVD) in Colombia by department, 2014–2017. Departments are ordered from North to South down the columns. Y axes are different for each plot. Fig C. City elevation. Comparison of elevation (in meters) between cities that were invaded versus cities that escaped invasion for (a) CHIKV, (b) ZIKV, and (c) CHIKV, ZIKV, or DENV. Fig D. Example of algorithm used to estimate invasion week using the generation time method. (a) The time series for Caucasia in the department of Antioquia during the 2015–2017 ZIKV epidemic. In this figure, week 1 corresponds to the week ending on August 15, 2015, and week 51 corresponds to the week ending on July 30, 2016. The algorithm identifies the point of maximum incidence in the time series and counts backward one week at a time until there are no reported cases. If there are no cases in this week or the prior two or three weeks depending on the infection’s generation time, then this is the invasion week. If not, the algorithm continues to go back in time until the condition is met. The part of the line in red is the period used to determine the onset of invasion, and the blue dashed line is the estimated invasion week. (b) The same time series as in (a) is shown until the point of maximum incidence (week ending on January 23, 2016). The estimated time of invasion is week 15 rather than week 21 because cases were reported in weeks 16–20. Fig E. Comparison of estimated invasion weeks using two methods. A method based on the first reported cases in each city (x-axis) and a method based on the generation time distribution of each infection (generation time method, y-axis) were compared for (a) CHIKV and (b) ZIKV. The black line is y = x. The two methods show good agreement (CHIKV: r = 0.60, ZIKV: r = 0.68). Fig F. Comparison [file pcbi.1009174.s001.docx]

**Spatial and temporal invasion dynamics of the 2014-2017**

**Zika and chikungunya epidemics in Colombia**

**S1 Text**

Kelly Charniga^1^*, Zulma M. Cucunubá^1^, Marcela Mercado^2^, Franklyn Prieto^2^, Martha Ospina^2^, Pierre Nouvellet^3^, Christl A. Donnelly^1,4^

^1^ Medical Research Council Centre for Global Infectious Disease Analysis, Department of Infectious Disease Epidemiology, Imperial College London, London, United Kingdom

^2^ Instituto Nacional de Salud, Bogotá, Colombia

^3^ School of Life Sciences, University of Sussex, Brighton, United Kingdom

^4^ Department of Statistics, University of Oxford, Oxford, United Kingdom

**Data for main analysis**

**Epidemiological data.** The two main datasets for this analysis consist of anonymized line lists on 105,152 Zika virus (ZIKV) disease and 411,789 chikungunya fever suspected and laboratory-confirmed cases with non-missing city (administrative level 2) location reported to Sivigila, Colombia’s national public health surveillance system, between 2014 and 2017. Fig A shows the epidemiological curves for chikungunya fever, ZIKV disease, and dengue fever at the country level, and Fig B shows the epidemiological curves for chikungunya fever and ZIKV disease at the department level (administrative level 1). Information related to public health events in the national territory is generated from the local levels by local health service providers. There are about 14,000 institutional, municipal, departmental, or national reporting bodies in Colombia. Chikungunya fever was added to the list of notifiable conditions in 2014, and ZIKV disease was added in 2015. Each week these data are aggregated and published [1].

The line list data were aggregated at administrative level 2 (municipalities), which we denote as “cities.” The location used in this analysis is the location of likely infection, which is determined by the clinician who reported the case. This location is preferred over residence because it accounts for human movement within Colombia as some areas are higher risk than others. In the ZIKV dataset, out of 105,152 cases, 93.2% (97,962) had matching administrative level 2 locations of likely infection and residence, meaning most people were infected where they live. Three-thousand and eighty-six cases (3.3%) resided in Bogotá, and 3,630 cases (3.5%) had different but valid within-country administrative level 2 locations. These cases were likely infected while traveling to other Colombian cities. Only 74 cases (0.07%) had another country listed as their residence, including the US, Peru, France, Italy, Switzerland, Portugal, and Venezuela. While some of these travelers could have introduced ZIKV into Colombia, we cannot exclude the possibility that they were infected while visiting.

Data were further aggregated by week based on either date of symptom onset or date of notification. For chikungunya virus (CHIKV), this resulted in a 110-week interval, from the week ending June 7, 2014 to that ending July 9, 2016, and for ZIKV, a 97-week interval, from the week ending August 15, 2015 to that ending June 17, 2017 (main text, Fig 3).

Population demographic data are from 2016 and were obtained from DANE, Colombia’s National Administrative Department of Statistics. These include population projections for 2016 derived from the 2005 Census. Population sizes were re-scaled by dividing by 10,000. For cities included in the main analysis, population sizes ranged from 1,670 to about 2.4 million. Latitudes and longitudes corresponding to the geographic center of each city were provided by our collaborators at Colombia’s Instituto Nacional de Salud (INS) and were used to calculate distances between cities. Here, geographic distance between cities is the geodesic distance calculated using the Vincenty inverse formula for ellipsoids using the gdist function in the Imap package (version 1.32) in R [2].

**Human mobility proxy.** Travel time between cities: 2015 data on accessibility to cities in Colombia were obtained from the Malaria Atlas Project [3]. The friction surface consists of average land-based travel speed, and units are in minutes to travel 1 meter. The costDistance function in the R package gdistance (version 1.2-2) was used to calculate the time to travel between cities in the country [4]. Two cities, San Andrés and Providencia, were not included in the dataset because they are islands and must be reached by air or sea. In order to compare models across distance metrics, these cities were dropped from preliminary analyses. Puerto Colombia in the department of Atlántico was also missing from this dataset, but this city is not an island. Because it shares a large border with Barranquilla, we used the same values for both cities, and the time to travel between them was assumed to be 0 minutes. The final models using geographic distance include all invaded cities and are presented in the results of the main text.

**Data for analysis of invasion risk factors**

**Epidemiological data.** We obtained data from Sivigila on 647,665 cases of dengue fever reported between 2010 and 2016 to analyze risk factors of CHIKV and ZIKV invasion.

**Elevation.** SRTM 90m Digital Elevation Data for Colombia were downloaded from the CGIAR-CSI GeoPortal, and the elevation of each city was extracted according to its latitude and longitude [5]. To better approximate human risk of vector-borne disease, coordinates corresponding to the population weighted centroids were used for most cities (n = 1,047). Due to incomplete country shapefiles, however, geographic center was used for some locations (n = 75).

**Weather data.** Weekly mean temperature data for Colombia from January 1, 2014 to October 1, 2016 were downloaded from Dryad Digital Repository [6]. These data were weighted by the population and aggregated at administrative levels 1 and 2. However, ZIKV disease cases were reported through mid-2017. To obtain temperature data after October 1, 2016, daily meteorological station readings were downloaded from the National Oceanic and Atmospheric Administration (NOAA)’s Climate Data Online [7]. This website contains past weather and climate data that are publicly and freely available to download.

PERSIANN-Cloud Classification System (PERSIANN-CCS) satellite precipitation data for Colombia were downloaded at daily time steps from the Center for Hydrometeorology and Remote Sensing (CHRS) Data Portal from January 1, 2014 to December 31, 2017 [8].

WorldPop Project data were downloaded from [6] as the weighting variable for the spatial aggregation of weather covariates. These data consist of 2015 estimates of the number of people per pixel with national totals adjusted to match the United Nations Population Division estimates. They were subset and resampled by Siraj et al. to match other climate variables considered in their database (~93 m resolution) [9].

**Socioeconomic data.** Multidimensional poverty data for Colombia were downloaded from DANE at the city level for the year 2018 [10]. The source of information for the calculation of multidimensional poverty at the department level is the Encuesta Nacional de Calidad de Vida (Quality of Life Survey). The annual Quality of Life Survey gathers information about Colombians’ living and housing conditions. DANE used information collected from the 2018 Censo Nacional de Población y Vivienda (the Census) to approximate multidimensional poverty at the city level. The calculations are based on households that were effectively censused.

Three variables were extracted from the multidimensional poverty data that might be relevant for arbovirus transmission, including the percentage of houses in the city with overcrowded conditions, inadequate exterior walls, and inadequate floors. Overcrowding was defined in terms of the number of people sleeping per room excluding the kitchen, bathroom, and garage and including the living room and dining room. A house was considered overcrowded if there were three or more people sleeping per room in an urban area and at least four people per room in a rural area. In an urban area, exterior walls made from unfinished wood, boards, planks, bamboo or other vegetation, zinc, fabric, and cardboard were defined as inadequate, along with damaged walls or no walls. In a rural area, walls constructed of bamboo or other vegetation, zinc, fabric, and cardboard were considered inadequate as well as damaged walls or no walls. Inadequate floors were those that were made of dirt in both urban and rural areas.

## Analyses of elevation, invasion weeks, long-distance transmission events, and infectivity

**Elevation.** In Colombia, one of the primary vectors of CHIKV and ZIKV, *Aedes aegypti*, is not typically found at elevations above 2,200 m due to environmental factors, especially temperature. Hence, cities located at high elevations have less risk of invasion [11,12]. Although evidence suggests that the distribution of mosquitoes occurs across altitudinal gradients in South America [13], we did not include the effects of city altitude on invasion spread in our models. Elevation is likely a proxy for whether a city becomes invaded rather than when. If there were an association between city elevation and time of invasion, there may not have been enough variation in elevation to explain disease spread. The models only included cities that reported epidemics, almost all of which are located in low-lying areas (under 2,200 m). Although Rees et al. found that mean city elevation significantly decreased the time to the first reported ZIKV case in Colombia using accelerated failure time models, the effect size was small, and they assumed all cities could become infected [14].

In Fig C, we compared the elevation of cities that were invaded versus cities that escaped invasion for (a) CHIKV, (b) ZIKV, and (c) CHIKV, ZIKV, or dengue virus (DENV). DENV, which causes dengue fever, is spread by the same species of mosquitoes as CHIKV and ZIKV and is endemic in Colombia. Invasion for CHIKV and ZIKV was defined as in the main text: cities that reported at least 20 cases of chikungunya fever were considered to have been invaded by CHIKV, and cities that reported at least 30 cases of ZIKV disease were considered to have been invaded by ZIKV. Invasion by DENV was defined as cities with at least 98 reported cases of dengue fever between 2010 and 2016. Ninety-eight is the median number of cases reported across all cities with any cases during this time period. This cut-off was used because over 700 cities reported at least 30 cases between 2010 and 2016.

The number of cities invaded by any of the three arboviruses (n=591) corresponds well with a 2013 report by Colombia’s Ministry of Health (MOH), which classified 56 cities as hyperendemic for DENV and 575 cities as mesoendemic [15]. The MOH criteria for level of DENV endemicity included trends of reported cases over time, number of circulating serotypes, age range of cases, and the presence of dengue hemorrhagic fever (severe dengue).

Cities that met the criteria for invasion typically had elevations below 2,000 m. Using the Wilcoxon rank sum test, the difference in elevation between invaded cities and uninvaded cities was statistically significant for each of the three sets of comparisons in Fig C (p < 0.0001).

**Determining invasion week by generation time method rather than using first reported cases.** We explored an alternative way of determining invasion weeks using weekly time series of chikungunya fever and ZIKV disease cases. For each disease and city separately, the first week of maximum incidence was identified. We then considered the time point just before this week (t_max – 1_). If zero cases were reported in this week and in the preceding two or three weeks, for CHIKV and ZIKV respectively, then this was the estimated invasion week. If cases were reported during this time period, we continued counting backwards one week at a time until the condition was met. The two and three weeks correspond to each infection’s generation time, the average time between the time of infection in a primary case and the time of infection in a secondary case infected by the primary case [16,17]. If there are no cases reported during this time period, then there is no evidence that transmission is occurring, and the disease is not yet established in that place assuming complete reporting.

Fig D shows an example of this algorithm for determining invasion week in each city, and Fig E shows the correlation between this method and the method using first reported cases as in the main text.

**Comparison of invasion week methods.** Methods for estimating invasion weeks have been developed previously for influenza [18,19]. A key feature of these methods is determining when influenza illnesses exceed baseline disease levels due to seasonal influenza. In Colombia, there were no baseline levels of chikungunya fever or ZIKV disease prior to 2014. Thus, even small numbers of cases are potentially interesting. Many of the epidemic curves at the city level are jagged with cases rising and falling repeatedly. The proposed methods here have advantages over existing methods because invasion weeks tend to be placed more frequently before these irregular spikes in cases. Also, vector biology is accounted for in the generation time.

Fig F compares invasion weeks estimated using our generation time method and a method from Charu et al. using linear piecewise splines. There is very good agreement between the two.

**Long-distance transmission events.** The number and locations of transmission events that occurred over long distances for CHIKV and ZIKV were determined. We used the minimum distance between a newly invaded city and cities invaded earlier to create a distribution (d-D) and classified cities invaded in the 99^th^ percentile as being invaded via long-distance events. Fig P shows the distribution of (d-D) for CHIKV and ZIKV graphically, and Table C shows summary statistics of this distribution. Tables D-E show the recipient and potential source cities of the long-distance transmission events.

**Infectivity matrix.** Weekly case counts were weighted by the generation time distributions of CHIKV and ZIKV using the R package EpiEstim (version 1.1-2) [20]. Values for the mean and standard deviation of the generation time distributions used for the weighting are shown in Table F.

**Movies.** S1 and S2 Movies show monthly incidence of chikungunya fever and ZIKV disease, respectively, per 100,000 population on a hexagonal grid. The geogrid package (version 0.1.1) in R was used to create a hexagonal grid of Colombia from shapefiles using the Hungarian algorithm [21].

**Risk factors of invasion**

**Data processing steps.** Mean temperature: the data processing workflow from Siraj et al. [9] was used to generate the mean temperature across Colombia from January 1, 2016 through January 31, 2017. In general, spatial models (kriging) were used on readings from meteorological stations in Colombia. A model is needed to interpolate the data over the parts of the country that do not have stations. This particular model with altitude and secondary temperature data as covariates was selected over (i) kriging without covariates and (ii) non-parametric surface fitting with thin-plate splines with or without covariates, based on leave-one-out cross validation. After kriging, the data were rasterized. Daily gridded data were generated from the raster files, which were then aggregated by week and multiplied by the population.

Siraj et al. used the first version of NOAA’s Climate Data Online tool, the Legacy Climate Data Online [22]. From the Global Summary of the Day data product, they extracted the minimum daily temperature, maximum daily temperature, mean daily temperature, and relative humidity from 30 stations between January 1, 2016 and December 31, 2016. The dataset containing mean temperature that they downloaded for 2016 was obtained from the GitHub repository linked to their paper (<https://github.com/asiraj-nd/zika-colombia>) as well as the Global 30 Arc-Second Elevation dataset, the WorldClim dataset, and NOAA’s Climate Prediction Center surface air temperature dataset.

To obtain 2017 station readings, we selected the Daily Summaries data product from the current version of NOAA’s Climate Data Online tool [7]. Five stations were missing from the new data. However, these stations had relatively few observations in the data originally downloaded by Siraj et al.; the five stations had between seven and 362 observations over three years (1,096 days) compared to the other 25 stations, which had between 1,078 and 1,096 observations. The 2_KRG_predict_tmean.R file in the above GitHub repository was used to perform the kriging on the mean temperature data for 2016 and 2017, resulting in one .bil file for each day. These files were read back into R as rasters. The daily data were aggregated at weekly time steps by taking the average of each consecutive seven raster layers. Next, the WorldPop layer was subset and resampled to match the spatial extent and resolution of the temperature layers (4.65 km x 4.65 km resolution). The weekly temperature layers were then multiplied by the WorldPop layer as in [9] in order to weight the rasters by the human population. The resulting weekly layers and the WorldPop layer were exported to Python (version 3.8.2).

In Python, the QgsZonalStatistics.Sum function from the qgis.analysis module was used to spatially aggregate the temperature data. This function calculates the sum of the raster values for a polygon and appends the results as attributes. Administrative level 2 shapefiles for 2018 were obtained from Colombia’s Sistema de Información Geográfica para la Planeación y el Ordenamiento Terriorial [23]. After re-projecting the shapefiles from Mercator to WSG84, spatial aggregation by summing was performed on the weekly temperature layers that had been multiplied by the WorldPop layer as well as the WorldPop layer. The resulting shapefiles were re-imported into R, where the aggregated mean temperature values were divided by the aggregated population values.

The new 2016-2017 population weighted weekly time series of mean temperature was compared to the 2014-October 2016 data from Siraj et al. There was good agreement for 2016, and seasonal trends were consistent in the 2017 data. The final temperature dataset uses the mean temperature from Siraj et al. for 2014-October 2016 and the newly processed data for the remaining two months of 2016 and 2017.

Precipitation: daily satellite precipitation data were read into R as rasters. Missing values were re-coded from -99 to 0. As with the temperature data, the spatial extent and resolution of the WorldPop layer was subset and resampled to match the precipitation data (4 km x 4 km resolution). Next, the daily data were aggregated at weekly time steps by taking the sum of each consecutive seven raster layers, resulting in cumulative precipitation for each week (if the missing values are not re-coded, then negative rainfall can be obtained for some locations and weeks as a result of this step). These layers were multiplied by the WorldPop layer and were exported to Python.

Spatial aggregation of the precipitation data was performed with the same shapefiles and Python code as the temperature data. The final precipitation dataset consists of only the population weighted weekly time series generated from the PERSIANN-CCS data. Although the new data overlapped well with the corresponding time series from Siraj et al., the latter dataset contained one outlier for the city of San Andrés (915 mm on the week ending July 23, 2016) and two outliers for the department of San Andrés and Providencia (771 mm and 823 mm on the weeks ending on August 30, 2014 and July 23, 2016, respectively) that could not be explained by extreme weather events such as hurricanes.

**Results.** Results of the univariate analyses and best-fitting logistic regression models can be found in Tables G-J. Fig G shows the distribution of invasion weeks according to dengue risk level.

## Spatial transmission model diagnostics and validation

**Model convergence.** For each model, we ran three Markov chain Monte Carlo (MCMC) chains from three different starting points to ascertain convergence. Figs H-I show the MCMC traces for each best-fitting gravity model including the burn-in period. It does not take long for the chains to move toward the same values and overlap with each other. Table A shows the Gelman-Rubin statistic for each of the best-fitting gravity models (after removing the burn-in). All point estimates and 95% CI bounds are one or approximately one, suggesting model convergence. Table B shows the acceptance rates of parameters for the same models, both of which have good acceptance rates.

**Choice of thresholds for invasion.** Table K shows the estimated parameters of gravity models fitted to different numbers of cities using thresholds of 10, 20, and 30 cumulative reported cases. In each case, the best-fitting model was the infectivity model with the μ parameter set to 0. All credible intervals overlap, indicating the model results are robust to the choice of threshold. Fig J shows the results of the corresponding epidemic simulations.

**Simulation study.** Results of the simulation study can be found in Table L. For each virus, all of the credible intervals of the estimated parameters from the observed data and the simulated data overlap, confirming that we were able to recover the fitted parameter estimates from the best-fitting gravity models.

**Single-introduction assumption.** Fig K shows the results of relaxing the single-introduction assumption for the best-fitting CHIKV gravity model.

**Gravity model variants.** Two distance variants were tested for each model type: geographic distance and travel time between cities. Tables M-P show results of four gravity model variants using 337 and 287 cities for CHIKV and ZIKV, respectively. One less city is modeled for each virus compared to the best-fitting individual models used for gravity model diagnostics and validation due to missing estimated land-based travel times for the island of San Andrés. Log transforming travel time between cities did not improve model DIC. The best-fitting models for ZIKV according to DIC are those incorporating geographic distance, while CHIKV model variants performed similarly across distance metrics.

**Models fitted jointly to arboviruses.** Table Q shows a comparison of alternative models of CHIKV and ZIKV spread in Colombia. The models are ordered by sum of DIC and were fitted to each virus independently. The individual and joint models for the two best-fitting model variants are presented in the main text. Table R shows the parameter estimates and DIC values of the third and fourth best-fitting individual and joint model variants.

**Best-fitting individual models used for model diagnostics and validation.** The best-fitting models that were independently fitted to each epidemic are shown in Tables S-T. These models were fitted to 338 and 288 cities for CHIKV and ZIKV, respectively, as geographic distance data were available for all invaded cities. The infectivity model with μ set to 0 was used for model diagnostics and validation as it had the lowest sum of DIC across arboviruses. We also tested a version of this model that incorporated per capita infectivity rather than infectivity, but the DICs were higher (indicating worse fit) for both CHIKV and ZIKV (ΔDIC of 9.5 for CHIKV and ΔDIC of 4 for ZIKV).

**Gravity model fits using estimated invasion week by generation time method.** Figs L-M show the probability distributions of invasion weeks and epidemic simulations using the generation time method of estimating invasion week. The same thresholds for number of reported cases were used as in the main text, resulting in 338 cities for CHIKV and 288 cities for ZIKV. Using geographic distance, the best-fitting model for CHIKV was the infectivity model (estimating six parameters), whereas the best-fitting ZIKV model was the one that estimated density dependence with the power for susceptible population size set to 0 (estimated β, γ, ν, and ε). Parameter estimates (Figs S-T) were similar to those presented in Tables S-T and discussed in the main text.

Using these estimates of invasion weeks, the CHIKV model fitted the observed data less well. One possible explanation is that this method for determining invasion weeks is more sensitive to lower reporting at the beginning of the CHIKV epidemic compared to the first reported cases method. When the first cases were identified in Colombia in June 2014, chikungunya fever was not yet a notifiable disease, and therefore, some cities reported all of their cases retrospectively. One city reported nearly 1,400 cases in a single week. This could have led the model to underestimate the infection pressure early in the epidemic.

**Gravity model fits using first reported cases and all cities.** We also fitted models to all 1,122 cities in Colombia. As in the main text, the probability that a susceptible city *j* is invaded at time *t_j_* was

$$P(t_{j})=exp\left( -\sum_{\tau=0}^{t_{j}-1} \lambda_{j,\tau} \right)\left( 1-exp\left( -\lambda_{j,t_{j}} \right) \right)$$

The probability that a city escaped invasion was

$$P(t_{j})=exp\left( -\sum_{\tau=0}^{T} \lambda_{j,\tau} \right)$$

where the force of infection for city *j* is summed over the entire epidemic (from week 0 to T). Fig N shows epidemic simulations using the first reported cases method of estimating invasion week and all cities in Colombia. The best-fitting model for CHIKV estimated the same five parameters as in the main text, whereas the best-fitting model for ZIKV estimated all six parameters. This result is presented for illustrative purposes only. The mathematical modeling framework used in this study allows us to explain disease spread, not lack of spread. Similar studies in the literature did not include all cities in the countries that they modeled. For example, Gog et al. included 271 cities for the US, and Eggo et al. included 47 cities for the US and 246 cities for England and Wales. Charu et al. included a range from 135 to 306 U.S. cities [18,19,24].

**Probability distribution of first reported cases.** Figs Q-R show the probability distribution of invasion week by department. As in the main text, this calculation was performed using the median parameter estimates from the posterior distribution for the best-fitting gravity models. Cities that fall outside of the central 95% interval of their expected distribution tended to be invaded at the beginning or the end of the epidemics.

**Fig A. Weekly reported cases of chikungunya fever (CF), dengue fever (DF), and Zika virus disease (ZVD) in Colombia, January 2010 – June 2017.**

**Fig B. Epidemiological curves of chikungunya fever (CF) and Zika virus disease (ZVD) cases in Colombia by department, 2014-2017.** Departments are ordered from North to South down the columns. Y axes are different for each plot.

**Fig C. City elevation.** Comparison of elevation (in meters) between cities that were invaded versus cities that escaped invasion for (a) CHIKV, (b) ZIKV, and (c) CHIKV, ZIKV, or DENV.

Fig D. Example of algorithm used to estimate invasion week using the generation time method. (a) The time series for Caucasia in the department of Antioquia during the 2015-2017 ZIKV epidemic. In this figure, week 1 corresponds to the week ending on August 15, 2015, and week 51 corresponds to the week ending on July 30, 2016. The algorithm identifies the point of maximum incidence in the time series and counts backward one week at a time until there are no reported cases. If there are no cases in this week or the prior two or three weeks depending on the infection’s generation time, then this is the invasion week. If not, the algorithm continues to go back in time until the condition is met. The part of the line in red is the period used to determine the onset of invasion, and the blue dashed line is the estimated invasion week. (b) The same time series as in (a) is shown until the point of maximum incidence (week ending on January 23, 2016). The estimated time of invasion is week 15 rather than week 21 because cases were reported in weeks 16-20.

Fig E. Comparison of estimated invasion weeks using two methods. A method based on the first reported cases in each city (x-axis) and a method based on the generation time distribution of each infection (generation time method, y-axis) were compared for (a) CHIKV and (b) ZIKV. The black line is y = x. The two methods show good agreement (CHIKV: r = 0.60, ZIKV: r = 0.68).

Fig F. Comparison of estimated invasion weeks using two methods. A method based on the generation time distribution (generation time method, x-axis) and a piecewise spline method (Charu method, as in [24], y-axis) were compared for (a) CHIKV and (b) ZIKV. 95% confidence intervals are shown for the Charu method only. For some cities, only the point estimate for *t_j_* fell within the 95% confidence interval; this is shown by a lack of vertical bar. The two methods show very good agreement (CHIKV: r = 0.90, ZIKV: r = 0.70).

**Fig G. Distribution of invasion week by dengue risk level for (a) CHIKV and (b) ZIKV.** The black lines are the fitted linear regression models.


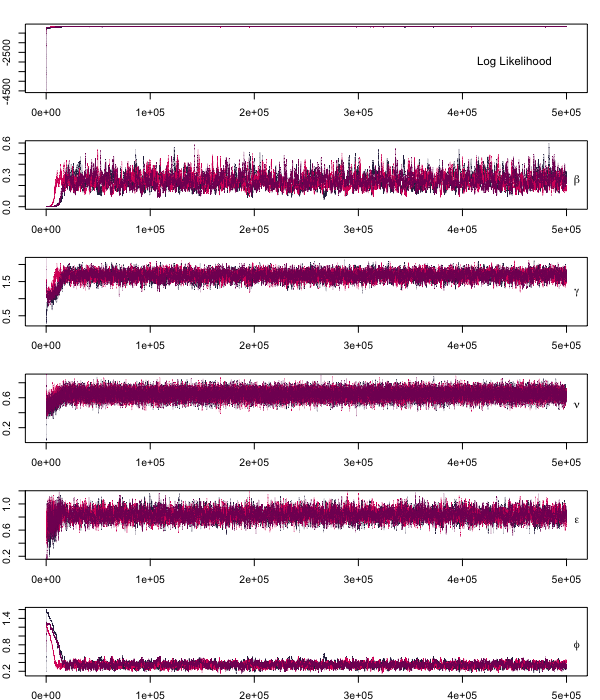


**Fig H. MCMC chains for the best-fitting CHIKV gravity model from three different starting points.**

**
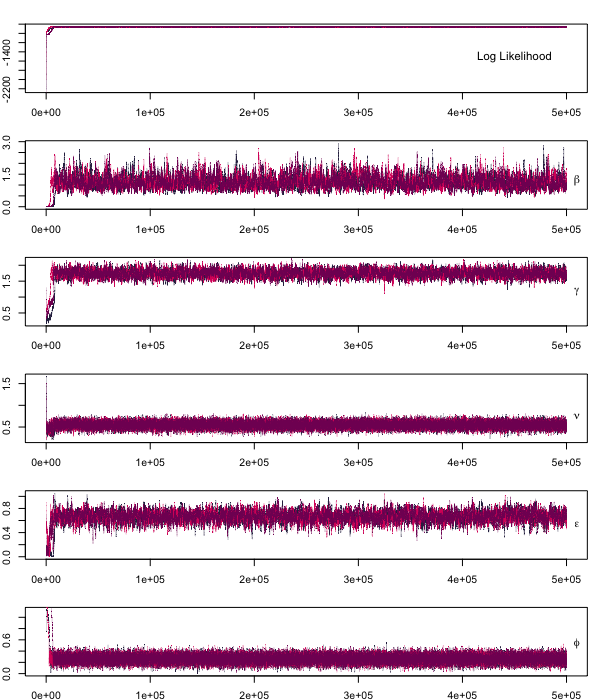
**

**Fig I. MCMC chains for the best-fitting ZIKV gravity model from three different starting points.**

**Fig J. Epidemic simulations of the best-fitting gravity models showing the sensitivity of the thresholds used to determine invasion.**

**Fig K. Comparison of the distance kernel obtained when running the CHIKV gravity model from week 12 versus the entire dataset.** The distance power estimates were similar when parameter estimation started from week 12 (1.77 [95% CrI: 1.54-1.99]) compared to week 1 (1.68 [95% CrI: 1.44-1.90]).


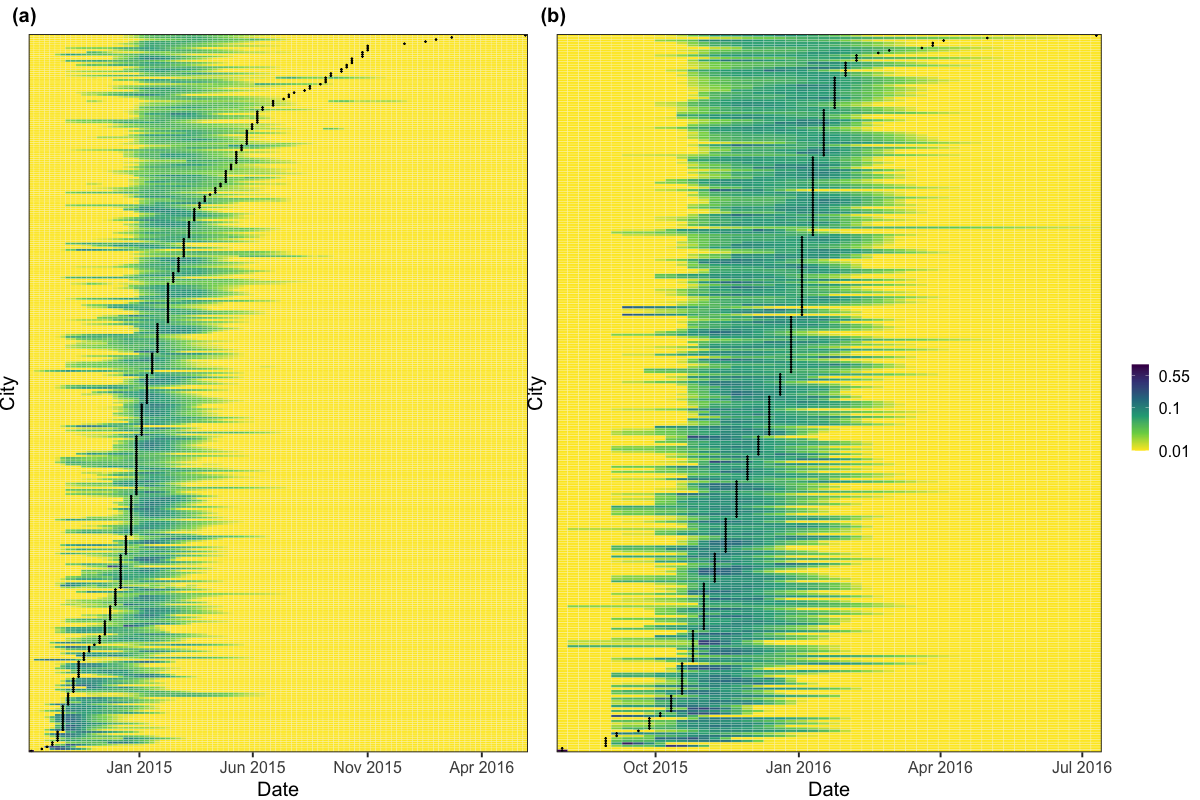


**Fig L. Probability distribution of estimated invasion week (generation time method).** Colored lines represent the probability distribution of invasion week for (a) CHIKV and (b) ZIKV. The calculations were performed using the median parameter estimates from the posterior distributions of the models using estimated invasion week rather than first reported cases. The black lines show the estimated invasion week in each city using a method based on each infection’s generation time. Values of 0.01 represent probabilities of 0.01 or less.

Fig M. Epidemic invasion simulations (generation time method). Simulated invasion for (a) CHIKV and (b) ZIKV from the models using estimated invasion week by generation time method rather than week of first reported cases. Simulated epidemics are shown in light gray. The dark gray lines are the average across the 1,000 simulations. The red lines are the observed incidence curves.

**Fig N. Epidemic invasion simulations (all cities).** Simulated invasion for (a) CHIKV and (b) ZIKV from the models using week of first reported cases and all 1,122 cities in Colombia. Simulated epidemics are shown in light gray. The dark gray lines are the average across the 1,000 simulations. The red lines are the observed incidence curves.

**Fig O. Epidemic invasion simulations (best-fitting Stouffer’s rank models).** Results correspond to the models presented in Tables S-T. Simulated invasion for (a) CHIKV and (b) ZIKV from the models using week of first reported cases. Simulated epidemics are shown in light gray. The dark gray lines are the average across the 1,000 simulations. The red lines are the observed incidence curves.

Fig P. Long-distance transmission events. The distribution of d-D for (a) CHIKV and (b) ZIKV in this study. The dashed blue lines are plotted at the 97.5^th^ percentile (corresponding to 212.00 km and 255.33 km for CHIKV and ZIKV, respectively) and the dashed red lines are plotted at the 99^th^ percentile (corresponding to 344.40 km and 321.21 km for CHIKV and ZIKV, respectively). Long-distance transmission events were defined as invasions that occurred in cities included in the 99^th^ percentile of this distribution.


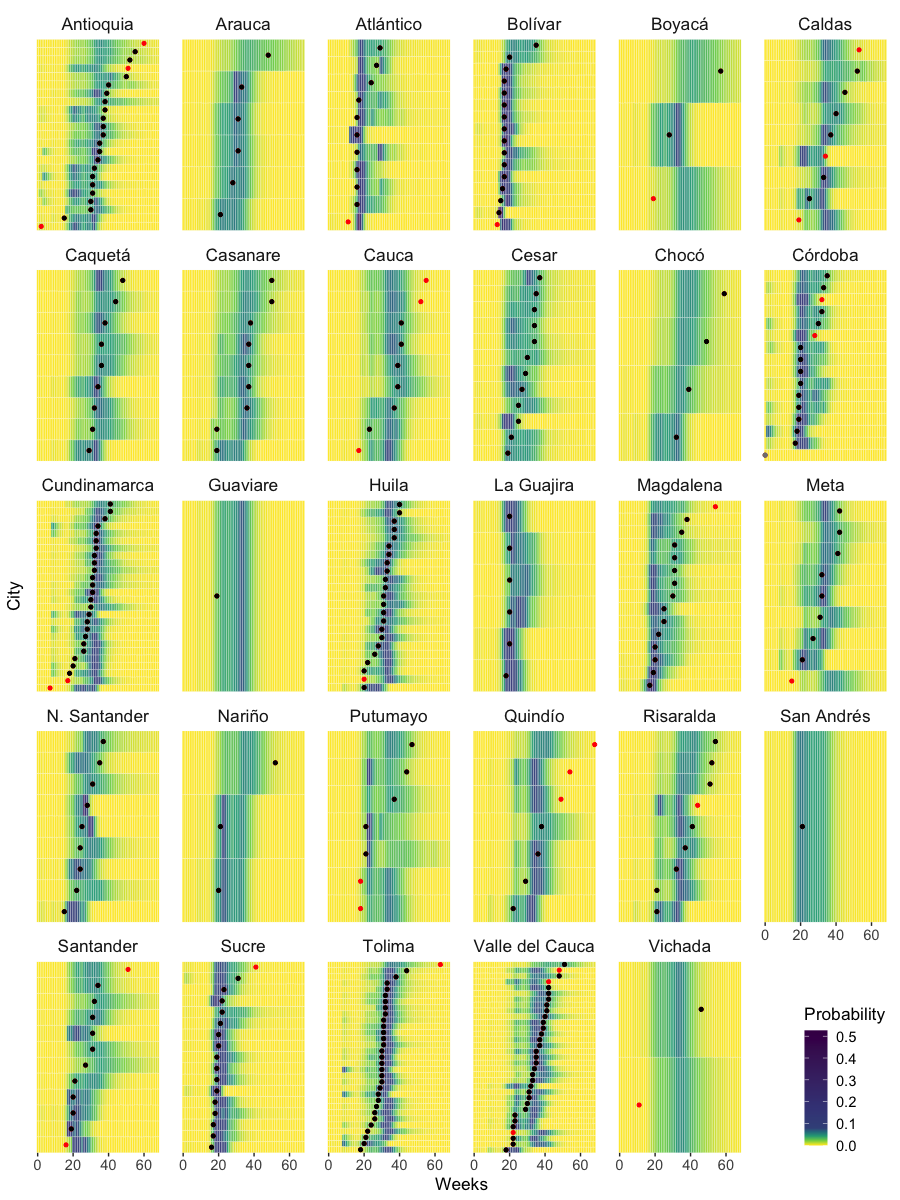


Fig Q. Probability distribution of first reported cases by department for CHIKV. Black circles are cities that fall within the 95% interval of their expected distribution, and red circles fall outside this interval. The gray circle in the department of Córdoba represents the city that was invaded in week 0.


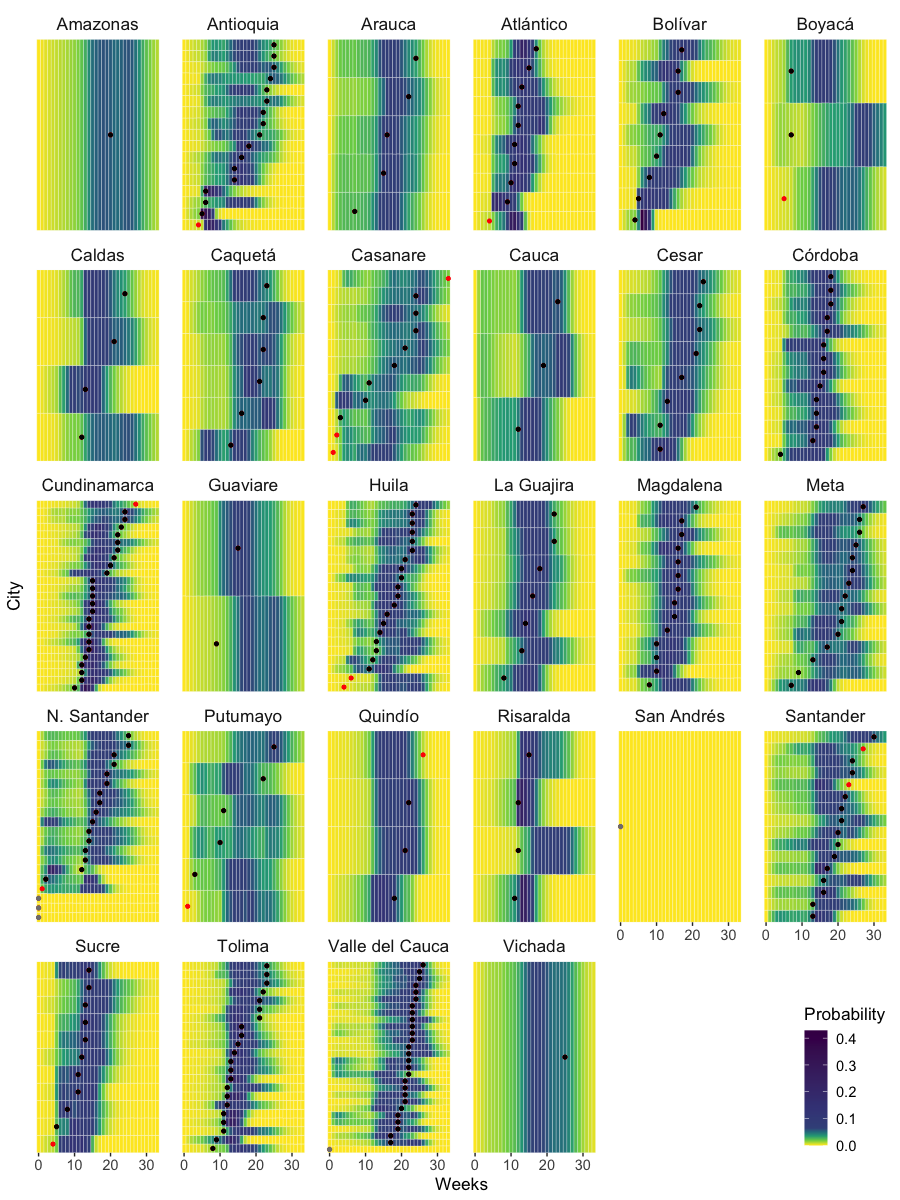


Fig R. Probability distribution of first reported cases by department for ZIKV. Black circles are cities that fall within the 95% interval of their expected distribution, and red circles fall outside this interval. The gray circles in the departments of San Andrés, Valle del Cauca, and Norte de Santander represent cities that were invaded in week 0.

Fig S. Parameter estimates for CHIKV model fitted using estimated invasion week by generation time method. The dashed red line shows the median of the posterior distribution of each parameter after removing the burn-in period. The blue line shows the median of the posterior distribution from the model fitted using the method based on first reported cases as in the main text. Only parameters that are estimated in both models are shown.

Fig T. Parameter estimates for ZIKV model fitted using estimated invasion week by generation time method. The dashed red line shows the median of the posterior distribution of each parameter after removing the burn-in period. The blue line shows the median of the posterior distribution from the model fitted using the method based on first reported cases as in the main text. Only parameters that are estimated in both models are shown.

Table A. Gelman-Rubin statistic for each of the best-fitting gravity models (after removing the burn-in).

|  | CHIKV | | ZIKV | |
| --- | --- | --- | --- | --- |
|  | Point estimate | Upper CI | Point estimate | Upper CI |
| γ (distance power) | 1 | 1.00 | 1 | 1.00 |
| ν (invaded population) | 1 | 1.00 | 1 | 1.00 |
| ε (spatial interaction) | 1 | 1.01 | 1 | 1.00 |
| φ (infectivity) | 1 | 1.00 | 1 | 1.00 |
| **β (intensity)** | 1 | 1.00 | 1 | 1.00 |

Table B. Acceptance percentages for parameters of the best-fitting CHIKV and ZIKV gravity models.

| Parameter | CHIKV | ZIKV |
| --- | --- | --- |
| γ (distance power) | 20.3 | 25.6 |
| ν (invaded population) | 22.4 | 26.8 |
| ε (spatial interaction) | 21.9 | 22.8 |
| φ (infectivity) | 19.5 | 27.3 |
| β (intensity) | 22.2 | 24.1 |

Table C. Summary statistics of the d-D distributions.

| (d-D) Summary Statistics | | | | | | | | | |
| --- | --- | --- | --- | --- | --- | --- | --- | --- | --- |
|  | **Min** | **1^st^ quartile** | **Median** | **Mean** | **3^rd^ quartile** | **Max** | **# cities** | **N>99^th^%** | **N>97.5^th^%** |
| CHIKV | 0 | 0 | 5.00 | 25.72 | 27.00 | 475 | 337* | 4 | 9 |
| ZIKV | 0 | 0 | 3.50 | 25.60 | 23.00 | 346 | 280** | 3 | 7 |

This table shows that ZIKV and CHIKV exhibited similar patterns of transmission. The first six columns have units in km. The seventh column is the total sample size, and the last two columns contain the number of long-distance transmission events for two distance thresholds.

*Cities invaded in week 0 were excluded.

**Cities invaded in weeks 0 and 1 were excluded as week 1 was the first week in which any cities were considered infectious.

**Table D. Recipient and potential source cities of long-distance transmission events of CHIKV.**

| **Recipient cities** | **Potential source cities** |
| --- | --- |
| Girardot, Cundinamarca | Apartadó, Antioquia  Planeta Rica, Córdoba |
| La Primavera, Vichada | Apartadó, Antioquia  Planeta Rica, Córdoba  Girardot, Cundinamarca |
| Mocoa, Putumayo and  Puerto Asís, Putumayo | Apartadó, Antioquia  Envigado, Antioquia  Barranquilla, Atlántico  Baranoa, Atlántico  Campo de la Cruz, Atlántico  Sabanalarga, Atlántico  Santo Tomás, Atlántico  Soledad, Atlántico  Suan, Atlántico  Turbaco, Bolívar  Cartagena, Bolívar  San Juan Nepomuceno, Bolívar  Santa Rosa, Bolívar  Planeta Rica, Córdoba  Girardot, Cundinamarca  Barranca de Upía, Meta  Cúcuta, Norte de Santander  Floridablanca, Santander  Corozal, Sucre  La Primavera, Vichada |

**Table E.** **Recipient and potential source cities of long-distance transmission events of ZIKV.**

| **Recipient cities** | **Potential source cities** |
| --- | --- |
| Tauramena, Casanare | Cúcuta, Norte de Santander  El Zulia, Norte de Santander  Puerto Santander, Norte de Santander  San Andrés, San Andrés and Providencia  Cali, Valle del Cauca |
| Barranquilla, Atlántico and  Cartagena, Bolívar | Aguazul, Casanare  Tauramena, Casanare  Cúcuta, Norte de Santander  El Zulia, Norte de Santander  Puerto Santander, Norte de Santander  Ocaña, Norte de Santander  Villa del Rosario, Norte de Santander  Mocoa, Putumayo  San Andrés, San Andrés and Providencia  Cali, Valle del Cauca |

**Table F. Estimates of the mean and standard deviation of the generation time distribution.**

|  | **Mean** | **Standard Deviation** | **Reference** |
| --- | --- | --- | --- |
| CHIKV generation time | 14.0 | 6.2 | [17] |
| ZIKV generation time | 20.0 | 7.4 | [16] |

Estimates were used to calculate city infectivity. All values have units in days.

**Table G. Univariate analysis of risk factors of CHIKV invasion.**

|  | Invaded cities (N = 338)* | | Uninvaded cities (N = 784)** | |  |
| --- | --- | --- | --- | --- | --- |
|  | Mean | SD | Mean | SD | P |
| Population size, thousands | 72 | 180 | 31 | 300 | <0.0001 |
| Elevation, m | 585 | 592 | 1,461 | 1,045 | <0.0001 |
| Mean study period temperature, °C | 25.5 | 3.3 | 20.5 | 5.8 | <0.0001 |
| Mean temperature up to epidemic peak, °C | 25.1 | 3.3 | 19.9 | 5.9 | <0.0001 |
| Mean study period rainfall, mm | 48.3 | 27.4 | 44.4 | 29.1 | 0.0003 |
| Mean rainfall up to epidemic peak, mm | 49.5 | 31.6 | 43.3 | 31.8 | <0.0001 |
| Percentage of households with overcrowding | 12.3 | 7.0 | 10.7 | 7.6 | <0.0001 |
| Percentage of households with inadequate exterior walls | 6.1 | 7.1 | 4.8 | 8.7 | <0.0001 |
| Percentage of households with inadequate floors | 14.2 | 15.5 | 16.3 | 17.3 | 0.10 |
| Mean travel time, min. | 736 | 224 | 935 | 1,118 | 0.87 |
| Dengue risk, No. (%) |  |  |  |  |  |
| 0 | 11 (3.3) |  | 326 (41.6) |  | <0.0001 |
| 1 | 38 (11.2) |  | 224 (28.6) |  |  |
| 2 | 105 (31.1) |  | 157 (20.0) |  |  |
| 3 | 184 (54.4) |  | 77 (9.8) |  |  |

*337 cities were included in the row for mean travel time.

**783 cities were included in the row for mean travel time.

**Table H. Univariate analysis of risk factors of ZIKV invasion.**

|  | Invaded cities (N = 288)* | | Uninvaded cities (N = 834)** | |  |
| --- | --- | --- | --- | --- | --- |
|  | Mean | SD | Mean | SD | P |
| Population size, thousands | 89 | 241 | 28 | 278 | <0.0001 |
| Elevation, m | 577 | 522 | 1,411 | 1,055 | <0.0001 |
| Mean study period temperature, °C | 25.4 | 3.2 | 20.9 | 5.7 | <0.0001 |
| Mean temperature up to epidemic peak, °C | 25.7 | 3.2 | 21.1 | 5.9 | <0.0001 |
| Mean study period rainfall, mm | 50.7 | 24.2 | 49.0 | 26.4 | 0.04 |
| Mean rainfall up to epidemic peak, mm | 40.3 | 26.4 | 40.1 | 26.7 | 0.36 |
| Percentage of households with overcrowding | 12.3 | 6.8 | 10.8 | 7.7 | <0.0001 |
| Percentage of households with inadequate exterior walls | 5.6 | 6.0 | 5.0 | 8.9 | <0.0001 |
| Percentage of households with inadequate floors | 13.5 | 14.5 | 16.5 | 17.5 | 0.06 |
| Mean travel time, min. | 769 | 600 | 912 | 1,038 | 0.86 |
| Dengue risk, No. (%) |  |  |  |  |  |
| 0 | 5 (1.7) |  | 332 (39.8) |  | <0.0001 |
| 1 | 21 (7.3) |  | 241 (28.9) |  |  |
| 2 | 81 (28.1) |  | 181 (21.7) |  |  |
| 3 | 181 (62.8) |  | 80 (9.6) |  |  |

*287 cities were included in the row for mean travel time.

**833 cities were included in the row for mean travel time.

**Table I. Best-fitting logistic regression model of CHIKV invasion.**

|  | **Estimate** | **Std. error** | **Z value** | **P value** | **Odds ratio (95% CI)** |
| --- | --- | --- | --- | --- | --- |
| Intercept | -5.75 | 0.55 | -10.52 | <0.0001 | 0.003  (0.001-0.009) |
| Mean study period temperature, °C | 0.22 | 0.03 | 7.46 | <0.0001 | 1.24  (1.17-1.32) |
| Mean study period rainfall, 10 mm | -0.18 | 0.04 | -5.00 | <0.0001 | 0.84  (0.78-0.90) |
| Dengue tertile 1 (ref 0) | 0.58 | 0.41 | 1.42 | 0.16 | 1.78  (0.82-4.07) |
| Dengue tertile 2 (ref 0) | 1.58 | 0.40 | 3.99 | <0.0001 | 4.85  (2.30-10.97) |
| Dengue tertile 3 (ref 0) | 2.74 | 0.39 | 6.97 | <0.0001 | 15.50  (7.39-34.84) |
| Mean travel time, hr | -0.06 | 0.02 | -3.32 | 0.0009 | 0.94  (0.91-0.97) |

Models were fitted to 1,120 cities because mean travel time between cities was not available for two island cities.

**Table J. Best-fitting logistic regression model of ZIKV invasion.**

|  | **Estimate** | **Std. error** | **Z value** | **P value** | **Odds ratio (95% CI)** |
| --- | --- | --- | --- | --- | --- |
| Intercept | -0.96 | 0.68 | -1.42 | 0.16 | 0.38  (0.09-1.38) |
| Elevation, 100 m | -0.11 | 0.02 | -5.58 | <0.0001 | 0.90  (0.86-0.93) |
| Mean study period rainfall, 10 mm | -0.21 | 0.04 | -4.99 | <0.0001 | 0.81  (0.74-0.88) |
| Percentage of households with inadequate exterior walls | -0.03 | 0.01 | -2.58 | 0.01 | 0.97  (0.94-0.99) |
| Dengue tertile 1 (ref 0) | 0.68 | 0.57 | 1.20 | 0.23 | 1.98  (0.69-6.61) |
| Dengue tertile 2 (ref 0) | 2.30 | 0.54 | 4.27 | <0.0001 | 9.96  (3.73-31.9) |
| Dengue tertile 3 (ref 0) | 3.75 | 0.54 | 6.99 | <0.0001 | 42.3  (16.0-135.4) |

Models were fitted to all 1,122 cities in Colombia.

**Table K. Comparison of parameter estimates from gravity models fitted to different numbers of cities using thresholds of 10, 20, and 30 cumulative reported cases.**

|  | CHIKV | | | ZIKV | | |
| --- | --- | --- | --- | --- | --- | --- |
|  | Threshold of 30 reported cases | **Threshold of 20 reported cases** | Threshold of 10 reported cases | **Threshold of 30 reported cases** | Threshold of 20 reported cases | Threshold of 10 reported cases |
| Number of cities | 317 | **338** | 379 | **288** | 360 | 485 |
| γ (distance power) | 1.67  (1.43-1.90) | **1.68**  **(1.44-1.90)** | 1.64  (1.41-1.85) | **1.74**  **(1.51-1.96)** | 1.85  (1.64-2.08) | 1.93  (1.73-2.12) |
| μ (susceptible population) | 0 | **0** | 0 | **0** | 0 | 0 |
| ν (invaded population) | 0.64  (0.52-0.75) | **0.65**  **(0.53-0.76)** | 0.57  (0.46-0.67) | **0.55**  **(0.41-0.69)** | 0.60  (0.49-0.72) | 0.47  (0.38-0.56) |
| ε (spatial interaction) | 0.86  (0.72-1.02) | **0.83**  **(0.69-0.98)** | 0.75  (0.59-0.89) | **0.68**  **(0.50-0.84)** | 0.70  (0.56-0.83) | 0.70  (0.57-0.80) |
| φ (infectivity) | 0.32  (0.22-0.42) | **0.35**  **(0.25-0.48)** | 0.35  (0.25-0.49) | **0.27**  **(0.13-0.40)** | 0.32  (0.20-0.44) | 0.27  (0.16-0.37) |
| β (intensity) | 0.25  (0.16-0.40) | **0.24**  **(0.13-0.39)** | 0.25  (0.13-0.42) | **1.10**  **(0.68-1.77)** | 1.20  (0.78-1.88) | 1.20  (0.83-1.72) |

In each case, the model is the infectivity model with μ set to 0. Columns in bold correspond to results presented in the main text.

**Table L. Comparison of parameter estimates from observed data versus simulated data.**

|  | CHIKV | | ZIKV | |
| --- | --- | --- | --- | --- |
| Parameters | Estimates from observed data | Estimates from simulated data | Estimates from observed data | Estimates from simulated data |
| γ (distance power) | 1.68  (1.44-1.90) | 1.61  (1.40-1.81) | 1.74  (1.51-1.96) | 1.80  (1.58-2.03) |
| μ (susceptible population) | 0 | 0 | 0 | 0 |
| ν (invaded population) | 0.65  (0.53-0.76) | 0.76  (0.65-0.87) | 0.55  (0.41-0.69) | 0.47  (0.35-0.59) |
| ε (spatial interaction) | 0.83  (0.69-0.98) | 0.84  (0.68-1.00) | 0.68  (0.50-0.84) | 0.71  (0.54-0.88) |
| φ (infectivity) | 0.35  (0.25-0.48) | 0.31  (0.17-0.49) | 0.27  (0.13-0.40) | 0.29  (0.15-0.42) |
| β (intensity) | 0.24  (0.13-0.39) | 0.29  (0.14-0.50) | 1.10  (0.68-1.77) | 1.09  (0.64-1.79) |

**Table M. Parameter estimates for six gravity models of CHIKV for 337 cities using geographic distance.**

|  | Distance-only model | Density-dependent population model | Density-independent population model | Estimated density dependence population model | Full (infectivity) model | **Infectivity model with μ set to 0*** |
| --- | --- | --- | --- | --- | --- | --- |
| DIC | 2511.7 | 2440.7 | 2397.7 | 2396.9 | 2330.2 | **2329.4** |
| γ (distance power) | 1.05  (0.87-1.21) | 1.02  (0.81-1.19) | 1.46  (1.22-1.69) | 1.49  (1.25-1.73) | 1.68  (1.43-1.91) | **1.68**  **(1.44-1.90)** |
| μ (susceptible population) | 0 | 0.081  (0.004-0.328) | 0.037  (0.001-0.164) | 0.034  (0.002-0.146) | 0.060  (0.002-0.201) | **0** |
| ν (invaded population) | 0 | 0.45  (0.34-0.55) | 0.53  (0.42-0.64) | 0.53  (0.41-0.64) | 0.64  (0.52-0.76) | **0.65**  **(0.53-0.76)** |
| ε (spatial interaction) | 0 | 0 | 1 | 0.85  (0.66-1.03) | 0.83  (0.68-0.98) | **0.83**  **(0.68-0.99)** |
| φ (infectivity) | 0 | 0 | 0 | 0 | 0.35  (0.25-0.47) | **0.35**  **(0.25-0.46)** |
| β (intensity) | 0.16  (0.07-0.36) | 0.080  (0.023-0.207) | 0.26  (0.20-0.31) | 0.31  (0.22-0.44) | 0.21  (0.11-0.36) | **0.24**  **(0.14-0.39)** |

Posterior median and 95% credible interval presented for each parameter. Bold indicates the best-fitting model. Travel time data were only available for 337 out of 338 cities. To compare across distance metrics, 337 cities were also used for geographic distance models.

*When μ is set to 0, this means that cities with large populations have the same risk of being invaded as cities with small populations.

**Table N. Parameter estimates for six gravity models of CHIKV for 337 cities using travel time between cities.**

|  | Distance-only model | Density-dependent population model | Density-independent population model | Estimated density dependence population model | Full (infectivity) model | **Infectivity model with μ set to 0*** |
| --- | --- | --- | --- | --- | --- | --- |
| DIC | 2518.9 | 2459.4 | 2403.5 | 2395.9 | 2328.3 | **2326.9** |
| γ (distance power) | 0.89  (0.71-1.06) | 0.72  (0.53-0.90) | 1.66  (1.37-1.96) | 1.74  (1.45-2.03) | 1.95  (1.66-2.24) | **1.97**  **(1.69-2.25)** |
| μ (susceptible population) | 0 | 0.22  (0.01-0.50) | 0.042  (0.002-0.187) | 0.027  (0.001-0.127) | 0.054  (0.002-0.203) | **0** |
| ν (invaded population) | 0 | 0.40  (0.30-0.51) | 0.49  (0.38-0.60) | 0.48  (0.37-0.59) | 0.56  (0.45-0.67) | **0.57**  **(0.45-0.68)** |
| ε (spatial interaction) | 0 | 0 | 1 | 0.85  (0.75-0.94) | 0.79  (0.69-0.87) | **0.79**  **(0.70-0.87)** |
| φ (infectivity) | 0 | 0 | 0 | 0 | 0.39  (0.28-0.53) | **0.39**  **(0.28-0.52)** |
| β (intensity) | 0.11  (0.04-0.28) | 0.019  (0.005-0.067) | 0.24  (0.18-0.29) | 0.41  (0.26-0.62) | 0.30  (0.13-0.59) | **0.36**  **(0.19-0.65)** |

Posterior median and 95% credible interval presented for each parameter. Bold indicates the best-fitting model. Travel time data were only available for 337 out of 338 cities.

*When μ is set to 0, this means that cities with large populations have the same risk of being invaded as cities with small populations.

**Table O. Parameter estimates for six gravity models of ZIKV for 287 cities using geographic distance.**

|  | Distance-only model | Density-dependent population model | Density-independent population model | Estimated density dependence population model | Full (infectivity) model | **Infectivity model with μ set to 0*** |
| --- | --- | --- | --- | --- | --- | --- |
| DIC | 1804.5 | 1764.7 | 1749.0 | 1737.9 | 1715.5 | **1715.5** |
| γ (distance power) | 1.21  (1.01-1.38) | 1.28  (1.10-1.44) | 1.55  (1.31-1.78) | 1.65  (1.43-1.87) | 1.74  (1.50-1.97) | **1.74**  **(1.50-1.97)** |
| μ (susceptible population) | 0 | 0.021  (0.001-0.093) | 0.026  (0.001-0.113) | 0.018  (0.001-0.084) | 0.053  (0.003-0.210) | **0** |
| ν (invaded population) | 0 | 0.37  (0.26-0.48) | 0.46  (0.33-0.59) | 0.47  (0.34-0.60) | 0.55  (0.41-0.69) | **0.55**  **(0.41-0.69)** |
| ε (spatial interaction) | 0 | 0 | 1 | 0.67  (0.48-0.86) | 0.68  (0.50-0.86) | **0.67**  **(0.50-0.83)** |
| φ (infectivity) | 0 | 0 | 0 | 0 | 0.29  (0.16-0.42) | **0.27**  **(0.13-0.40)** |
| β (intensity) | 0.73  (0.28-1.63) | 0.69  (0.28-1.47) | 0.40  (0.32-0.48) | 0.86  (0.51-1.39) | 0.93  (0.53-1.59) | **1.11**  **(0.68-1.81)** |

Posterior median and 95% credible interval presented for each parameter. Bold indicates the best-fitting model. Travel time data were only available for 287 out of 288 cities. To compare across distance metrics, 287 cities were also used for geographic distance models.

*When μ is set to 0, this means that cities with large populations have the same risk of being invaded as cities with small populations.

**Table P. Parameter estimates for six gravity models of ZIKV for 287 cities using travel time between cities.**

|  | Distance-only model | Density-dependent population model | Density-independent population model | Estimated density dependence population model | Full (infectivity) model | **Infectivity model with μ set to 0*** |
| --- | --- | --- | --- | --- | --- | --- |
| DIC | 1828.1 | 1803.8 | 1764.9 | 1753.2 | 1735.8 | **1734.4** |
| γ (distance power) | 0.79  (0.52-1.02) | 0.73  (0.44-0.97) | 1.85  (1.49-2.19) | 1.99  (1.66-2.31) | 2.05  (1.71-2.39) | **2.06**  **(1.73-2.40)** |
| μ (susceptible population) | 0 | 0.034  (0.002-0.133) | 0.026  (0.001-0.111) | 0.017  (0.001-0.081) | 0.080  (0.003-0.279) | **0** |
| ν (invaded population) | 0 | 0.29  (0.18-0.40) | 0.39  (0.27-0.52) | 0.41  (0.28-0.54) | 0.46  (0.32-0.59) | **0.46**  **(0.33-0.59)** |
| ε (spatial interaction) | 0 | 0 | 1 | 0.82  (0.73-0.91) | 0.82  (0.73-0.90) | **0.81**  **(0.71-0.90)** |
| φ (infectivity) | 0 | 0 | 0 | 0 | 0.27  (0.13-0.41) | **0.23**  **(0.11-0.36)** |
| β (intensity) | 0.12  (0.02-0.44) | 0.06  (0.01-0.23) | 0.36  (0.29-0.44) | 0.94  (0.57-1.55) | 0.91  (0.42-1.69) | **1.20**  **(0.72-1.97)** |

Posterior median and 95% credible interval presented for each parameter. Bold indicates the best-fitting model. Travel time data were only available for 287 out of 288 cities.

*When μ is set to 0, this means that cities with large populations have the same risk of being invaded as cities with small populations.

**Table Q. Comparison of alternative models of CHIKV and ZIKV spread in Colombia.**

|  | Model type* | Distance type** | DIC | Sum of DIC | γ (distance power) | μ (susceptible population)*** | ν (invaded population) | ε (spatial interaction) | φ (infectivity) | β (intensity) |
| --- | --- | --- | --- | --- | --- | --- | --- | --- | --- | --- |
| CHIKV | G | GD | 2329.4 | 4044.9 | 1.68  (1.44-1.90) | 0 | 0.65  (0.53-0.76) | 0.83  (0.68-0.99) | 0.35  (0.25-0.46) | 0.24  (0.14-0.39) |
| ZIKV | G | GD | 1715.5 |  | 1.74  (1.50-1.97) | 0 | 0.55  (0.41-0.69) | 0.67  (0.50-0.83) | 0.27  (0.13-0.40) | 1.11  (0.68-1.81) |
| CHIKV | S | GD | 2322.9 | 4047.3 |  | 0.48  (0.37-0.58) | 1.18  (1.01-1.36) |  | 0.32  (0.24-0.42) | 0.009  (0.005-0.015) |
| ZIKV | S | GD | 1724.4 |  |  | 0.43  (0.31-0.55) | 1.37  (1.12-1.63) |  | 0.53  (0.44-0.63) | 0.021  (0.013-0.032) |
| CHIKV | S | TT | 2325.6 | 4054.8 |  | 0.47  (0.36-0.58) | 1.16  (0.99-1.35) |  | 0.31  (0.24-0.40) | 0.008  (0.005-0.013) |
| ZIKV | S | TT | 1729.2 |  |  | 0.42  (0.30-0.54) | 1.44  (1.15-1.79) |  | 0.48  (0.38-0.58) | 0.023  (0.013-0.037) |
| CHIKV | G | TT | 2326.9 | 4061.3 | 1.97  (1.69-2.25) | 0 | 0.57  (0.45-0.68) | 0.79  (0.70-0.87) | 0.39  (0.28-0.52) | 0.36  (0.19-0.65) |
| ZIKV | G | TT | 1734.4 |  | 2.06  (1.73-2.40) | 0 | 0.46  (0.33-0.59) | 0.81  (0.71-0.90) | 0.23  (0.11-0.36) | 1.20  (0.72-1.97) |
| CHIKV | SV | GD | 2333.5 | 4062.0 |  | 0.51  (0.40-0.61) | 1.17  (0.99-1.35) |  | 0.33  (0.25-0.41) | 0.009  (0.005-0.014) |
| ZIKV | SV | GD | 1728.5 |  |  | 0.46  (0.34-0.57) | 1.36  (1.10-1.66) |  | 0.54  (0.44-0.64) | 0.022  (0.013-0.035) |
| CHIKV | SV | TT | 2333.6 | 4070.4 |  | 0.51  (0.40-0.62) | 1.15  (0.98-1.35) |  | 0.33  (0.25-0.42) | 0.008  (0.004-0.014) |
| ZIKV | SV | TT | 1736.8 |  |  | 0.45  (0.33-0.57) | 1.41  (1.11-1.71) |  | 0.49  (0.40-0.59) | 0.022  (0.013-0.035) |
| CHIKV | RV | GD | 2427.3 | 4236.2 |  |  |  |  | 0.29  (0.24-0.36) | 0.034  (0.025-0.043) |
| ZIKV | RV | GD | 1808.9 |  |  |  |  |  | 0.65  (0.56-0.73) | 0.034  (0.026-0.044) |
| CHIKV | RV | TT | 2421.7 | 4240.0 |  |  |  |  | 0.30  (0.24-0.36) | 0.029  (0.021-0.037) |
| ZIKV | RV | TT | 1818.3 |  |  |  |  |  | 0.61  (0.52-0.69) | 0.032  (0.025-0.040) |
| CHIKV | R | GD | 2432.9 | 4246.9 |  |  |  |  | 0.30  (0.24-0.36) | 0.030  (0.023-0.038) |
| ZIKV | R | GD | 1814.0 |  |  |  |  |  | 0.65  (0.56-0.74) | 0.032  (0.024-0.040) |
| CHIKV | R | TT | 2431.3 | 4253.6 |  |  |  |  | 0.30  (0.24-0.37) | 0.025  (0.019-0.033) |
| ZIKV | R | TT | 1822.3 |  |  |  |  |  | 0.61  (0.52-0.70) | 0.029  (0.023-0.036) |

Posterior median and 95% credible interval presented for each parameter. Models are ordered by sum of DIC and were fitted separately to 337 cities for CHIKV and 287 cities for ZIKV.

*G: gravity (competing destinations), S: Stouffer’s rank, SV: Stouffer’s rank variant, R: radiation, RV: radiation variant

**GD: geographic distance, TT: travel time between cities

***When μ is set to 0, this means that cities with large populations have the same risk of being invaded as cities with small populations.

**Table R. Comparison of individual versus joint models of CHIKV and ZIKV spread in Colombia (third and fourth best-fitting model variants).**

|  | Model type* | Distance type** | DIC | Sum of DIC | | γ (distance power) | μ (susceptible population)*** | ν (invaded population) | ε (spatial interaction) | φ_a_ (infectivity) | φ_b_ (infectivity) | β_a_ (intensity) | β_b_ (intensity) |
| --- | --- | --- | --- | --- | --- | --- | --- | --- | --- | --- | --- | --- | --- |
| CHIKV | S | TT | 2325.6 | 4054.8 | |  | 0.47  (0.36-0.58) | 1.16  (0.99-1.35) |  | 0.31  (0.24-0.40) |  | 0.008  (0.005-0.013) |  |
| ZIKV | S | TT | 1729.2 |  |  |  | 0.42  (0.30-0.54) | 1.44  (1.15-1.79) |  | 0.48  (0.38-0.58) |  | 0.023  (0.013-0.037) |  |
| Joint | S | TT | 4145.1 | | |  | 0.43  (0.35-0.50) | 1.32  (1.16-1.47) |  | 0.20  (0.16-0.24) |  | 0.020  (0.015-0.026) |  |
| Joint | S | TT | 4060.7 | | |  | 0.44  (0.36-0.51) | 1.18  (1.05-1.33) |  | 0.39  (0.32-0.46) |  | 0.006  (0.004-0.010) | 0.019  (0.013-0.026) |
| Joint | S | TT | 4054.8 | | |  | 0.44  (0.37-0.52) | 1.23  (1.08-1.39) |  | 0.30  (0.23-0.39) | 0.49  (0.39-0.59) | 0.010  (0.006-0.015) | 0.016  (0.011-0.023) |
| CHIKV | G | TT | 2326.9 | | 4061.3 | 1.97  (1.69-2.25) | 0 | 0.57  (0.45-0.68) | 0.79  (0.70-0.87) | 0.39  (0.28-0.52) |  | 0.36  (0.19-0.65) |  |
| ZIKV | G | TT | 1734.4 | |  | 2.06  (1.73-2.40) | 0 | 0.46  (0.33-0.59) | 0.81  (0.71-0.90) | 0.23  (0.11-0.36) |  | 1.20  (0.72-1.97) |  |
| Joint | G | TT | 4141.9 | | | 2.02  (1.80-2.21) | 0 | 0.49  (0.40-0.57) | 0.84  (0.77-0.90) | 0.13  (0.08-0.17) |  | 0.72  (0.52-1.01) |  |
| Joint | G | TT | 4059.2 | | | 2.02  (1.82-2.24) | 0 | 0.52  (0.44-0.61) | 0.80  (0.75-0.86) | 0.32  (0.25-0.40) |  | 0.43  (0.30-0.63) | 1.20  (0.86-1.67) |
| Joint | G | TT | 4058.8 | | | 2.01  (1.76-2.21) | 0 | 0.52  (0.43-0.61) | 0.80  (0.74-0.86) | 0.38  (0.27-0.49) | 0.25  (0.12-0.37) | 0.35  (0.20-0.58) | 1.22  (0.86-1.75) |

Posterior median and 95% credible interval presented for each parameter. Models were fitted to 337 cities for CHIKV and 287 cities for ZIKV. For the joint models that estimate different parameters across arboviruses, parameters with subscript a refer to CHIKV, while parameters with subscript b refer to ZIKV.

*G: gravity (competing destinations), S: Stouffer’s rank

**TT: travel time between cities

***When μ is set to 0, this means that cities with large populations have the same risk of being invaded as cities with small populations.

**Table S.** **Parameter estimates for seven models of CHIKV in Colombia for 338 cities.**

|  | Distance-only model | Density-dependent population model | Density-independent population model | Estimated density dependence population model | Full (infectivity) model | **Infectivity model with μ set to 0*** | Stouffer’s rank model |
| --- | --- | --- | --- | --- | --- | --- | --- |
| DIC | 2521.0 | 2448.4 | 2403.0 | 2402.6 | 2336.1 | **2335.1** | 2328.3 |
| γ (distance power) | 1.03  (0.83-1.21) | 1.02  (0.84-1.17) | 1.46  (1.22-1.70) | 1.49  (1.25-1.72) | 1.68  (1.43-1.90) | **1.68**  **(1.44-1.90)** |  |
| μ (susceptible population) | 0 | 0.076  (0.003-0.286) | 0.036  (0.001-0.163) | 0.030  (0.001-0.141) | 0.061  (0.002-0.207) | **0** | 0.48  (0.37-0.58) |
| ν (invaded population) | 0 | 0.45  (0.34-0.55) | 0.54  (0.42-0.65) | 0.53  (0.41-0.64) | 0.64  (0.52-0.76) | **0.65**  **(0.53-0.76)** | 1.19  (1.01-1.36) |
| ε (spatial interaction) | 0 | 0 | 1 | 0.86  (0.68-1.05) | 0.84  (0.69-1.00) | **0.83**  **(0.69-0.98)** |  |
| φ (infectivity) | 0 | 0 | 0 | 0 | 0.34  (0.25-0.46) | **0.35**  **(0.25-0.48)** | 0.32  (0.24-0.41) |
| β (intensity) | 0.15  (0.05-0.37) | 0.08  (0.03-0.19) | 0.26  (0.20-0.31) | 0.30  (0.22-0.43) | 0.21  (0.11-0.35) | **0.24**  **(0.13-0.39)** | 0.009  (0.005-0.015) |

The first six models are variations of the gravity model. Posterior median and 95% credible interval presented for each parameter. Bold indicates the model used in the validations.

*When μ is set to 0, this means that cities with large populations have the same risk of being invaded as cities with small populations.

**Table T. Parameter estimates for seven models of ZIKV in Colombia for 288 cities.**

|  | Distance-only model | Density-dependent population model | Density-independent population model | Estimated density dependence population model | Full (infectivity) model | **Infectivity model with μ set to 0*** | Stouffer’s rank model |
| --- | --- | --- | --- | --- | --- | --- | --- |
| DIC | 1803.8 | 1764.4 | 1748.7 | 1737.6 | 1716.2 | **1715.3** | 1724.4 |
| γ (distance power) | 1.19  (0.96-1.36) | 1.27  (1.08-1.42) | 1.55  (1.31-1.78) | 1.66  (1.43-1.89) | 1.75  (1.49-1.99) | **1.74**  **(1.51-1.96)** |  |
| μ (susceptible population) | 0 | 0.021  (0.001-0.094) | 0.027  (0.001-0.113) | 0.020  (0.001-0.086) | 0.091  (0.005-0.264) | **0** | 0.43  (0.32-0.55) |
| ν (invaded population) | 0 | 0.37  (0.26-0.48) | 0.46  (0.3- 0.59) | 0.47  (0.34-0.60) | 0.55  (0.40-0.68) | **0.55**  **(0.41-0.69)** | 1.37  (1.15-1.65) |
| ε (spatial interaction) | 0 | 0 | 1 | 0.67  (0.47-0.84) | 0.69  (0.52-0.84) | **0.68**  **(0.50-0.84)** |  |
| φ (infectivity) | 0 | 0 | 0 | 0 | 0.30  (0.17-0.44) | **0.27**  **(0.13-0.40)** | 0.53  (0.43-0.63) |
| β (intensity) | 0.66  (0.22-1.53) | 0.65  (0.25-1.35) | 0.40  (0.32-0.48) | 0.84  (0.52-1.41) | 0.86  (0.42-1.52) | **1.10**  **(0.68-1.77)** | 0.021  (0.013-0.033) |

The first six models are variations of the gravity model. Posterior median and 95% credible interval presented for each parameter. Bold indicates the model used in the validations.

*When μ is set to 0, this means that cities with large populations have the same risk of being invaded as cities with small populations.

**References**

1. Pacheco O, Beltrán M, Nelson CA, Valencia D, Tolosa N, Farr SL, et al. Zika Virus Disease in Colombia — Preliminary Report. New England Journal of Medicine. 2016.

2. Wallace JR. R package Imap. 1.32 ed2010.

3. Weiss DJ, Nelson A, Gibson HS, Temperly WH, Peedell S. A global map of travel time to cities to assess inequalities in accessibility in 2015. Nature. 2018;553:333-6.

4. van Etten J. R package gdistance: Distances and Routes on Geographical Grids. 1.2-2 ed2018.

5. Jarvis A, Reuter HI, Nelson A, Guevara E. Hole-filled SRTM for the globe Version 4: CGIAR-CSI SRTM 90m Database; 2008 [Available from: <https://cgiarcsi.community/data/srtm-90m-digital-elevation-database-v4-1/>.

6. Siraj AS, Rodriguez-Barraquer I, Barker CM, Tejedor-Garavito N, Harding D, Lorton C, et al. Data from: Spatiotemporal incidence of Zika and associated environmental drivers for the 2015-2016 epidemic in Colombia. Dryad Digital Repository 2017.

7. National Oceanic and Atmospheric Organization. Climate Data Online Search 2020 [Available from: <https://www.ncdc.noaa.gov/cdo-web/search?datasetid=GHCND>.

8. Nguyen P, Shearer E, Tran H, Ombadi M, Hayatbini N, Palacios T, et al. The CHRS Data Portal, an easily accessible public repository for PERSIANN global satellite precipitation data. Nature Scientific Data. 2019;6:180296.

9. Siraj AS, Rodriguez-Barraquer I, Barker CM, Tejedor-Garavito N, Harding D, Lorton C. Spatiotemporal Incidence of Zika and Associated Environmental Drivers for the 2015-2016 Epidemic in Colombia. Nature Scientific Data. 2018;5(180073).

10. Estadística DANd. Medida de pobreza multidimensional municipal de fuente censal 2018 Bogotá2020 [Available from: <https://www.dane.gov.co/index.php/estadisticas-por-tema/pobreza-y-condiciones-de-vida/pobreza-y-desigualdad/medida-de-pobreza-multidimensional-de-fuente-censal>.

11. Suarez MF, Nelson MJ. Registro de Altitud de Aedes Aegypti en Colombia. Biomedica. 1981;1(4):225.

12. Watts AG, Miniota J, Joseph HA, Brady OJ, Kraemer MUG, Grills AW, et al. Elevation as a proxy for mosquito-borne zika virus transmission in the americas. PLoS ONE. 2017;12(5):e0178211.

13. Lippi CA, Stewart-Ibarra AM, Franklin Bajaña Loor ME, Dueñas Zambrano JE, Espinoza Lopez NA, Blackburn JK, et al. Geographic shifts in Aedes aegypti habitat suitability in Ecuador using larval surveillance data and ecological niche modeling: Implications of climate change for public health vector control. PLoS Neglected Tropical Diseases. 2019;13:e0007322.

14. Rees EE, Petukhova T, Mascarenhas M, Pelcat Y, Ogden NH. Environmental and social determinants of population vulnerability to Zika virus emergence at the local scale. Parasites & Vectors. 2018;11:290.

15. Colombia Ministerio de Salud y Protección Social. Situación Actual de Dengue a Semana 12 de 2013 Periodo de Análisis: 2008-2013 2013 [Available from: <https://www.minsalud.gov.co/Documentos%20y%20Publicaciones/INFORME%20SITUACION%20DE%20DENGUE.pdf>.

16. Ferguson NM, Cucunubá ZM, Dorigatti I, Nedjati-Gilani GL, Donnelly CA, Basánez M, et al. Countering Zika in Latin America. Science. 2016;353(6297):353-4.

17. Salje H, Lessler J, Kumar Paul K, Azman AS, Waliur Rahman M. How social structures, space, and behaviors shape the spread of infectious diseases using chikungunya as a case study. PNAS. 2016;113(47):13420-5.

18. Eggo RM, Cauchemez S, Ferguson NM. Spatial dynamics of the 1918 influenza pandemic in England, Wales and the United States. Journal of The Royal Society Interface. 2011;8(55):233-43.

19. Gog JR, Ballesteros S, Viboud C, Simonsen L, Bjornstad ON, Shaman J, et al. Spatial Transmission of 2009 Pandemic Influenza in the US. PLoS Computational Biology. 2014;10(6):e1003635.

20. Cori A, Cauchemez S, Ferguson NM, Fraser C, Dahlqwist E. R package EpiEstim: Estimate Time Varying Reproduction Numbers from Epidemic Curves. 1.1-2 ed2013.

21. Bailey J, Hafen R, Nowosad J, Zehnder L. R package geogrid: turn geospatial polygons into regular or hexagonal grids. 2018.

22. National Oceanic and Atmospheric Organization. NNDC Climate Data Online 2020 [Available from: <https://www7.ncdc.noaa.gov/CDO/cdo>.

23. Sistema de información geográfica para la planeación y el ordenamiento territorial. Visor SIG-OT Colombia 2018 [Available from: <https://sigot.igac.gov.co>.

24. Charu V, Zeger S, Bjørnstad ON, Kissler S, Simonsen L, Grenfell BT, et al. Human Mobility and the Spatial Transmission of Influenza in the United States. PLoS Computational Biology. 2017;13(2):e1005382.
