# Supplementary material for: Spatial and temporal invasion dynamics of the 2014–2017 Zika and chikungunya epidemics in Colombia
Source: PLoS Comput Biol. 2021 Jul 2;17(7):e1009174. doi: 10.1371/journal.pcbi.1009174 (PMC8291727; doi:10.1371/journal.pcbi.1009174)
Supplement: Attachment — Submitted filename: Comments .pdf [file pcbi.1009174.s004.pdf]

OVERVIEW

This manuscript made use of detailed epidemiological data to infer the spatiotemporal dynamics of CHIKV and ZIKV invasion in Colombia. Fitting gravity models to the week of invasion of CHIKV and ZIKV for different cities, the authors found that the spatial pattern of spread was explained using geographical distance and a model that accounted for density dependence. Moreover, they determined that the spatial pattern of spread was characterized by more frequent short-distance transmission events, rather than long-distance transmission events.

The manuscript provides a thorough analysis of the spatiotemporal patterns of spread for CHIKV and ZIKV. A more complete understanding of how infectious diseases invade a population could be important for planning of future outbreaks. As the authors note, the framework used in this manuscript could be used to forecast the future spread of infectious diseases, though some technical challenges need to be resolved.

I believe that this manuscript contributes to our spatiotemporal understanding of arboviruses, though certain methodological and analytical improvements could be made before publication. First, the manuscript could benefit from additional validation exercises of the inference framework, particularly with respect to the parameter estimates. Second, the analysis would be stronger – and the parameter estimates more robust – if the authors modeled not only the invasion of CHIKV and ZIKV into select cities but also the absence of invasion in the remaining cities. Finally, some of the interpretations of the effects of the susceptible population size on the timing of invasion may be over-stated, given the negligible differences between competing models on the basis of DIC.

SPECIFIC COMMENTS

[Lines 110-111] The authors estimated that the origin for both epidemics was Barranquilla. How does this compare to what would be obtained on the basis of line-list data alone? Are the first reported cases in Barranquilla? If so, how does the model provide information on the origin of the epidemic, beyond what could be obtained from the data alone?

[Lines 132-134] Which cities are the possible “sources” of the affected cities in each long-distance transmission event? How long are the distances, and how do these compare to the mean transmission distance?

[Lines 142-144] The models are fit to CHIKV and ZIKV separately. The authors note that both arboviruses are transmitted by the same vector and were introduced into an immunologically naïve population. Therefore, one might expect that the spatiotemporal patterns of spread would not be different across arboviruses. The authors should consider an additional analysis in which they fit the model jointly for CHIKV and ZIKV, assuming that the parameters are the same for both arboviruses. They also might consider variants of this, such as allowing the intensity parameter to vary across arboviruses. Comparison of all models could then be made on the basis of DIC.

[Lines 178-181] The authors note that cities invaded later by CHIKV were smaller in population. However, in lines 163-164, the authors contend that population size of the susceptible city appeared uncorrelated with invasion dynamics, and it seems that this claim was supported by the

best model having μ fixed at zero? How do the authors reconcile these two points? The delta DIC of the two best models was 0.9, which is below the threshold of 5-10 commonly used to identify the best-performing model. Might the claim that the model with μ fixed at zero is the best be overly strong in light of this and the observation that cities invaded late with CHIKV had smaller population sizes?

[Lines 202-203] I could not find the results for the analysis of the choice of cut-off in the Supporting Information. If these results are central to the methodological choices made in the manuscript, they should be included in the Supporting Information at a minimum.

[Lines 204-207] When you fit to all Colombian cities, the model cannot reproduce the distribution of invasion times for CHIKV and ZIKV (Fig. S7). Why is this the case? How are non-invaded cities accounted for in the model likelihood? If not already doing so, the authors could calculate the probability that each non-invaded city escaped invasion from t = 0 to the end of the epidemic time period. I think addressing the cities that were not invaded is important and presenting the analysis in which you only considered cities that were invaded could be misleading. The analysis should be capable of explaining both the invasion and lack of invasion of CHIKV and ZIKV in the country.

[Lines 245-250] Given the delta DIC of the two best performing models, it does not appear that the authors can make a claim one way or the other about the effects of the susceptible population size on invasion.

[Lines 298-307] Another potential challenge is that at the start of the epidemics, many CHIKV cases may have been misdiagnosed as DENV and many ZIKV cases may have been misdiagnosed as DENV and CHIKV. This could affect your estimate of the invasion week, given that weeks in which there were zero reported CHIKV cases, for instance, could have had DENV cases that were misdiagnosed and were in fact CHIKV. It appears that the authors analysis is robust to uncertainty surrounding the invasion week, but this is certainly a potential issue that may be worth exploring.

[Lines 314-320] Could the authors account for background importation rate of ZIKV and CHIKV, rather than assume that there was only a single introduction? A similar approach was taken by Guzzetta *et al.* (https://doi.org/10.1038/s41467-018-05230-4) when reconstructing transmission chains of DENV in Brazil. See the Supporting Information for how they integrated this into an analogous inference framework.

[Lines 341-342] This threshold simulation analysis should be included in the Supporting Information.

[Lines 362-366] I found the notation in these equations to be extremely confusing. Does t_k represent the timing of invasion of city k? Similarly, is t_j the timing of invasion of city j? Please clarify in the text.

[Lines 369-371 & 374-375] The minimum distance is calculated from pairwise distances. That assumes that the distance is a direct path from two cities. However, the authors may consider

calculating the shortest path from two cities on a graph where the weight of each edge is the distance metric considered. You could use the Floyd-Warshall Algorithm to get the all-pairs shortest paths. For geographical distance, the shortest path should likely just be the pairwise distance, but that may not be true for the travel times.

[Line 380] Did the authors consider other spatial models, such as the radiation model? If not, why?

[Lines 445-446] Convergence can be assessed using the Gelman-Rubin statistic, which provides a quantitative measure of convergence beyond visual inspection alone.

[Lines 453] The authors validate their estimates of the week of invasion by comparing the probability distribution obtained to the observed invasion weeks. However, no validation of the model parameters is done. The authors have a framework to simulate epidemics. They could therefore simulate synthetic data sets with the median parameter estimates that they obtained from the real data, and then re-run their analysis on this synthetic data set, confirming that they are able to re-obtain the fitted parameter estimates.
