# Supplementary material for: Spatial and temporal invasion dynamics of the 2014–2017 Zika and chikungunya epidemics in Colombia
Source: PLoS Comput Biol. 2021 Jul 2;17(7):e1009174. doi: 10.1371/journal.pcbi.1009174 (PMC8291727; doi:10.1371/journal.pcbi.1009174)
Supplement: Attachment — Submitted filename: Peer Review Response Letter FINAL.docx [file pcbi.1009174.s005.docx]

MRC Centre for Global Infectious Disease Analysis
Department of Infectious Disease Epidemiology
Imperial College London

St Mary’s Campus, Norfolk Place
London W2 1PG
United Kingdom

k.charniga17@imperial.ac.uk

22 February 2021	**Kelly Charniga BSc MPH**

Research Postgraduate

Sebastian Funk, PhD

Thomas Leitner, PhD

Editors

*PLOS Computational Biology*

Dear Professor Funk, Dr. Leitner, and Reviewers,

Thank you for sending the peer review comments on our manuscript entitled “**Spatial and temporal invasion dynamics of the 2014-2017 Zika and chikungunya epidemics in Colombia**.” We greatly appreciate the insights of the peer reviewers and have carefully considered each comment. We believe the paper is stronger as a result and hope you find the changes satisfactory.

In this letter, the line numbers from the peer review comments correspond to the first version of the paper, whereas the line numbers in our response correspond to the new version.

In response to code and data availability, we have made the code and data necessary to reproduce the best-fitting models and figures in the main text publicly available on GitHub <https://github.com/kcharniga/zika_chik_invasion>. We apologize for any confusion and have clarified the data availability statement in the paper (lines 673-675). The data and code files were provided to the journal as well as a README document explaining the data. The files are organized into folders on GitHub, but to make it easier for the peer reviewers, we have attached further instructions about which files are needed and how to run the code.

In response to Reviewer 2:

Comment 1: The authors must explain the use of the data of dengue fever cases mentioned in the supplementary material.

We have clarified in the supplement that the data on dengue fever cases were only used in the analyses on invasion risk factors (pg. 3). We apologize for not being clearer.

Comment 2: Does the invasion of ZIKV and CHIKV have any influence caused by the historical dengue virus transmission in the country? Do the hyperendemic cities for DENV have any influence on the delay of invasion by these arboviruses? Is there a possibility to discuss that?

We thank the reviewer for making this interesting point. Yes, we used linear regression models to test this. See lines 317-319 and 729-730 of the main text and S7 Fig in the supplement. We found that the time to invasion decreased by 3.4 (95% CI: 2.1-4.8) weeks for CHIKV and by 2.3 (95% CI: 1.3-3.4) weeks for ZIKV on average for each one-unit difference in dengue risk level.

Comment 3: It would be interesting to add a plot (perhaps on supplementary material) of weekly series of cases of ZIKV, CHIKV (and possibly DENV), for the period of study for the country level (or other appropriate regional divisions as desired by the authors).

We have added this plot to the supplement (S1 Fig).

Comment 4: Is there the possibility to combine Figure 3 in order to better visualize spatial and temporal occurrences of cases (incidence) of the diseases? Is there any situation of a peak of both diseases occurring at the same time and space?

We have added a plot of the epidemiological curves of ZIKV disease and chikungunya fever cases to the supplement. Departments are ordered from North to South (S2 Fig). We have also noted in the main text (lines 145-147) that Putumayo had epidemic peaks that were close together (eight weeks apart).

Comment 5: Reference is needed to foment the discussion presented from line 299 to 307. An example can be found in a recent work that analysed misclassification due to the simultaneous co-circulation of DENV, CHIKV, and ZIKV, in Brazil link <https://doi.org/10.1371/journal.pone.0228347> .

We would like to thank the reviewer for suggesting this reference and have now included it in the discussion (lines 464-475).

In response to Reviewer 1:

[Lines 110-111] The authors estimated that the origin for both epidemics was Barranquilla. How does this compare to what would be obtained on the basis of line-list data alone? Are the first reported cases in Barranquilla? If so, how does the model provide information on the origin of the epidemic, beyond what could be obtained from the data alone?

Based on the line list data, Barranquilla was not the first city in the country to report chikungunya fever or ZIKV disease cases. Barranquilla was among the first five cities to report cases of chikungunya fever, first reporting cases in week 12 (invaded in week 11). The city was also among the first 18 cities to report cases of ZIKV disease, first reporting cases in week 5 (invaded in week 4). Reporting at the beginning of the epidemics would have been unreliable because the diseases were new to the country, there were a high proportion of asymptomatic infections, and the presentation of symptoms is fairly unspecific. Therefore, the line list data alone are unlikely to be very useful for determining the geographic origin of the epidemics. We have clarified these points in the main text (lines 125-134 and 538-542) and apologize for any confusion.

[Lines 132-134] Which cities are the possible “sources” of the affected cities in each long- distance transmission event? How long are the distances, and how do these compare to the mean transmission distance?

We added two tables in the supplement (S4-S5) showing the recipient cities of long-distance transmission events in one column and the potential source cities in the second column. We also clarified in the main text how long these distances are and how they compare to the mean transmission distance (lines 165-168). We also updated S16 Fig in the supplement and the legend in Table 1 in the main text to correct an error found in the code for ZIKV.

[Lines 142-144] The models are fit to CHIKV and ZIKV separately. The authors note that both arboviruses are transmitted by the same vector and were introduced into an immunologically naïve population. Therefore, one might expect that the spatiotemporal patterns of spread would not be different across arboviruses. The authors should consider an additional analysis in which they fit the model jointly for CHIKV and ZIKV, assuming that the parameters are the same for both arboviruses. They also might consider variants of this, such as allowing the intensity parameter to vary across arboviruses. Comparison of all models could then be made on the basis of DIC.

We thank Reviewer 1 for this suggestion and have fitted gravity models and Stouffer’s rank models jointly for CHIKV and ZIKV. We found that the purely joint models which assumed the same parameters across arboviruses had worse fit than the individual models. However, joint models in which some parameters were allowed to vary across arboviruses had similar DIC values as the sum of the individual models’ DIC. The methods and results are described in lines 210-223 and 418-421 (also see Table 2), and more results can be found in the supplement (S18 Table).

[Lines 178-181] The authors note that cities invaded later by CHIKV were smaller in population. However, in lines 163-164, the authors contend that population size of the susceptible city appeared uncorrelated with invasion dynamics, and it seems that this claim was supported by $\mu$ fixed at zero? How do the authors reconcile these two points? The delta DIC of the two best models was 0.9, which is below the threshold of 5-10 commonly used to identify the best-performing model. Might the claim that the model with $\mu$ fixed at zero is the best be overly strong in light of this and the observation that cities invaded late with CHIKV had smaller population sizes?

We have added to lines 249-250 that the cities that were invaded late in the CHIKV epidemic represented only 3% of all invaded cities. In light of new results from considering other spatial models (namely Stouffer’s rank model), we have updated our conclusions about the effect of susceptible city population size (lines 388-390).

[Lines 202-203] I could not find the results for the analysis of the choice of cut-off in the Supporting Information. If these results are central to the methodological choices made in the manuscript, they should be included in the Supporting Information at a minimum.

The reviewer makes a valid point with this comment. We have added S11 Table and S10 Fig to the supplement showing the sensitivity of the model results to the choice of threshold for the number of reported cases. They show that our results are robust to the choice of threshold. Gravity model simulations were similar for thresholds of 10, 20, and 30 cases, and the credible intervals of all parameter estimates overlapped.

[Lines 204-207] When you fit to all Colombian cities, the model cannot reproduce the distribution of invasion times for CHIKV and ZIKV (Fig. S7). Why is this the case? How are non-invaded cities accounted for in the model likelihood? If not already doing so, the authors could calculate the probability that each non-invaded city escaped invasion from t = 0 to the end of the epidemic time period. I think addressing the cities that were not invaded is important and presenting the analysis in which you only considered cities that were invaded could be misleading. The analysis should be capable of explaining both the invasion and lack of invasion of CHIKV and ZIKV in the country.

See pg 8 of the supplement for the likelihood and further details. We also added an analysis of invasion risk factors which uses linear and logistic regression (lines 291-319 and 698-730 of the main text and pgs 4-7 of the supplement). Using multivariable logistic regression models, we found that mean rainfall and mean travel time were protective for CHIKV invasion, while mean temperature and dengue risk were associated with increased odds of invasion. For ZIKV, elevation, mean rainfall, and inadequate exterior walls were protective for invasion, while dengue risk was associated with increased odds of invasion. Using linear regression models, we also found that the time to invasion decreased by 3.4 (95% CI: 2.1-4.8) weeks for CHIKV and by 2.3 (95% CI: 1.3-3.4) weeks for ZIKV on average for each one-unit difference in dengue risk level.

[Lines 245-250] Given the delta DIC of the two best performing models, it does not appear that the authors can make a claim one way or the other about the effects of the susceptible population size on invasion.

As stated previously in our response to the comment about [Lines 178-181], we have added to lines 249-250 that the cities that were invaded at the end of the CHIKV epidemic represented only 3% of all invaded cities. In light of new results from considering other spatial models (namely Stouffer’s rank model), we have updated our conclusions about the effect of susceptible city population size (lines 388-390).

[Lines 298-307] Another potential challenge is that at the start of the epidemics, many CHIKV cases may have been misdiagnosed as DENV and many ZIKV cases may have been misdiagnosed as DENV and CHIKV. This could affect your estimate of the invasion week, given that weeks in which there were zero reported CHIKV cases, for instance, could have had DENV cases that were misdiagnosed and were in fact CHIKV. It appears that the authors analysis is robust to uncertainty surrounding the invasion week, but this is certainly a potential issue that may be worth exploring.

As stated in our response to Comment 5 from Reviewer 2, we have added a reference to this section (lines 464-475).

[Lines 314-320] Could the authors account for background importation rate of ZIKV and CHIKV, rather than assume that there was only a single introduction? A similar approach was taken by Guzzetta *et al.* (https://doi.org/10.1038/s41467-018-05230-4) when reconstructing transmission chains of DENV in Brazil. See the Supporting Information for how they integrated this into an analogous inference framework.

We thank the reviewer for this suggestion. Without additional data, actual importation of cases is difficult to characterize. There is little evidence that continued importations of CHIKV and ZIKV contributed to transmission in Colombia (see lines 496-503 and pg. 2 “Epidemiological data” in supplement). Regardless, we are more interested in the start of the epidemic in each city. For an epidemic to occur, a substantial number of cases are required to avoid stochastic extinction. Once we define the start of the epidemic in a city, the local dynamics would be more important than the contribution of importations given the low detection rate.

Furthermore, we agree that the single-introduction assumption should be tested and have done so for CHIKV in lines 280-284 of the main text with results in the supplement (S11 Fig). We found that when starting parameter estimation from week 12 of the epidemic, the estimate of the distance parameter was slightly higher. However, the credible intervals overlapped, suggesting that this assumption does not greatly affect the model fit.

[Lines 341-342] This threshold simulation analysis should be included in the Supporting Information.

As stated in our response to the previous comment regarding [Lines 202-203], our results are robust to the choice of threshold (see S11 Table and S10 Fig in the supplement).

[Lines 362-366] I found the notation in these equations to be extremely confusing. Does "! represent the timing of invasion of city *k*? Similarly, is " the timing of invasion of city *j*? Please clarify in the text.

We have clarified the notation in the equations (lines 554-555) and apologize for causing confusion.

[Lines 369-371 & 374-375] The minimum distance is calculated from pairwise distances. That assumes that the distance is a direct path from two cities. However, the authors may consider calculating the shortest path from two cities on a graph where the weight of each edge is the distance metric considered. You could use the Floyd-Warshall Algorithm to get the all-pairs shortest paths. For geographical distance, the shortest path should likely just be the pairwise distance, but that may not be true for the travel times.

Using the allShortestPaths function in the R package e1071 (version 1.7-4), we can confirm that for geographic distance, the shortest path is the pairwise distance. Compared to the long-distance transmission events determined using geographic distance, three out of seven cities were also identified as long-distance transmission events using travel times (Girardot, La Primavera, and Cartagena). However, we think it would be less confusing to readers to use only one type of distance metric in the descriptive analysis (geographic distance) and keep this section the way it is.

[Line 380] Did the authors consider other spatial models, such as the radiation model? If not, why?

We thank the reviewer for this important comment. We have now added to the analysis to include the radiation model and Stouffer’s rank model (lines 637-658 for methods).

[Lines 445-446] Convergence can be assessed using the Gelman-Rubin statistic, which provides a quantitative measure of convergence beyond visual inspection alone.

We have added the calculation of the Gelman-Rubin statistic (described in the main text in lines 666-670 and the results are in the supplement, S1 Table). We also added a section to the supplement on model convergence, including MCMC traces (S8-S9 Figs) and acceptance rates (S2 Table).

[Lines 453] The authors validate their estimates of the week of invasion by comparing the probability distribution obtained to the observed invasion weeks. However, no validation of the model parameters is done. The authors have a framework to simulate epidemics. They could therefore simulate synthetic data sets with the median parameter estimates that they obtained from the real data, and then re-run their analysis on this synthetic data set, confirming that they are able to re-obtain the fitted parameter estimates.

We performed the simulation study and added lines 273-275 and 692-695 in the main text and put the results in the supplement (S12 Table). We can confirm that we were able to recover the fitted parameter estimates after re-running the analysis on the synthetic dataset.

Thank you again for helping us to greatly improve our manuscript.

Sincerely,

Kelly Charniga
